# Innovation in the discovery of the HIV-1 attachment inhibitor temsavir and its phosphonooxymethyl prodrug fostemsavir

**DOI:** 10.1007/s00044-021-02787-6

**Published:** 2021-09-28

**Authors:** Tao Wang, John F. Kadow, Nicholas A. Meanwell

**Affiliations:** 1 Beijing Kawin Technology Share-Holdiing Co., 6 Rongjing East Street, BDA, Beijing, PR China; 2 ViiV Healthcare, 36 East Industrial Road, Branford, CT 06405 USA; 3 Small Molecule Drug Discovery, Bristol Myers Squibb Research and Early Development, P.O. Box 4000, Princeton, NJ 08543-4000 USA

**Keywords:** Fostemsavir, Indole-3-gyloxamide, HIV-1 attachment inhibitors, Prodrug, Synthetic methodology, Temsavir

## Abstract

The discovery and development of fostemsavir (**2**), the tromethamine salt of the phosphonooxymethyl prodrug of temsavir (**1**), encountered significant challenges at many points in the preclinical and clinical development program that, in many cases, stimulated the implementation of innovative solutions in order to enable further progression. In the preclinical program, a range of novel chemistry methodologies were developed during the course of the discovery effort that enabled a thorough examination and definition of the HIV-1 attachment inhibitor (AI) pharmacophore. These discoveries helped to address the challenges associated with realizing a molecule with all of the properties necessary to successfully advance through development and this aspect of the program is the major focus of this retrospective. Although challenges and innovation are not unusual in drug discovery and development programs, the HIV-1 AI program is noteworthy not only because of the serial nature of the challenges encountered along the development path, but also because it resulted in a compound that remains the first and only example of a mechanistically novel class of HIV-1 inhibitor that is proving to be very beneficial for controlling virus levels in highly treatment-experienced HIV-1 infected patients.

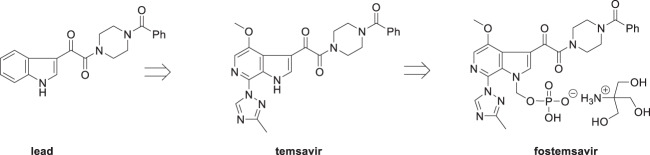

## Introduction

The human immunodeficiency virus-1 (HIV-1) attachment inhibitor (AI) fostemsavir (**2**), the tris(hydroxymethyl)methanamine (tris or tromethamine) salt of the phosphonooxymethyl prodrug of temsavir (**1**), was approved by the United States Food and Drug Administration (FDA) on July 1^st^, 2020 and by the European Medicines Agency (EMA) on February 9^th^, 2021 as a therapy for heavily treatment-experienced (HTE) patients infected with HIV-1 [1–3]. The discovery of temsavir (**1**) originated with a phenotypic screening assay that exploited a pseudotyped virus construct. The indole glyoxamide (**3**) was identified as an inhibitor of HIV-1 infection that was mechanistically novel, acting as an AI that interfered with the very first step of the virus entry process [4–6]. However, despite its relatively simple structure, the discovery of **3** subtended a twenty year odyssey that would lead to the commercialization of **2** [7]. The discovery and development of **1** and its transformation into **2** encountered significant challenges at almost every step of the preclinical and clinical programs; however, each of the obstacles encountered was met with a determined attitude that viewed each problem as an opportunity to devise and implement an innovative solution. While this is not atypical of successful projects in contemporary drug discovery and development within the pharmaceutical industry, in the case of **1** and **2** the innovation that was required was serial in nature. In this article we will describe the discovery of **1** and **2** against the backdrop of the development of new synthetic methodology that, while conceived to enable structure-activity exploration of these AIs, spawned additional avenues of innovative molecule assembly and manipulation. These discoveries emphasize the critical importance of the symbiotic relationship between organic chemistry and small molecule drug discovery that extends to embrace the meticulous design of molecules in order to explore structure-activity relationships (SARs) and address developability issues, all of which demand a deep and detailed understanding of the physicochemical attributes of heterocycles, individual functional groups and the complexities of their mutually dependent interactions [8–13].
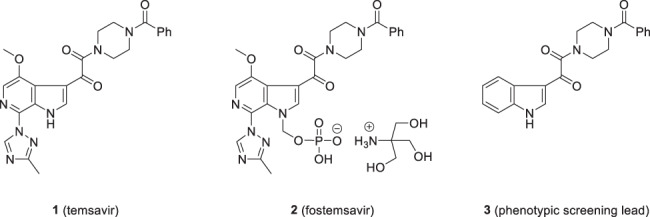


The lead glyoxamide **3** originated from a commercially available library of amides and sulfonamides that had developed some internal notoriety for the lack of structural integrity that had been encountered with many representatives. However, compound resynthesis, an essential first step in assessing high throughput screening leads, confirmed the identity of **3** and the purified material fully recapitulated the antiviral activity observed in the primary assay with the screening sample [5, 6]. When tested against a panel of available viruses, **3** was found to be a selective HIV-1 inhibitor, demonstrating no significant activity toward HIV-2, simian immunodeficiency virus (SIV), murine leukemia virus (MuLV), respiratory syncytial virus (RSV) influenza, Sendai virus, human cytomegalovirus (HCMV), vesicular stomatitis virus (VSV) and bovine viral diarrhea virus (BVDV) which, at the time, was being used as surrogate for hepatitis C virus (HCV). However, as a portent of one of the challenges that would be faced by the program in the later stages of discovery, the inhibitory potency of **3** toward a panel of 8 HIV-1 isolates that represented both macrophage-tropic (M-tropic) and T-cell (T-tropic) tropic viruses was somewhat variable, although there was no correlation with the identity of the co-receptor, either CCR5 or CXCR4 [5, 6]. Interest in **3** as a lead molecule was heightened considerably following mechanistic studies which ruled out allosteric inhibition of HIV-1 reverse transcriptase at the non-nucleoside binding site, well known for its promiscuity, as the source of the antiviral effect [14, 15]. This was indicative of a unique mode of action, with time-of-addition studies revealing that the molecule was acting early in the HIV-1 life cycle since the inhibitory efficacy declined as the interval between virus inoculation and compound introduction was increased [5]. When the HIV-1 envelope proteins in the pseudotyped virus were replaced with those from vesicular stomatitis virus (VSV), no inhibitory activity was observed, results that collectively were consistent with the HIV-1 envelope proteins being the biochemical target. Confirmation that the mechanism of inhibition by **3** was the virus entry process was obtained with a series of resistance selection experiments which identified Ala204Asp, Ala218Val and Phe423Tyr mutations in the HIV-1 gp120 spike protein as conferring reduced inhibitory sensitivity. These mutations mapped to regions within and proximal to the binding site of gp120 that recognizes Phe43 of CD4, the primary receptor on host cell membranes to which the virus attaches itself to initiate the cell entry process. An enzyme-linked immunosorbent assay (ELISA)-based biochemical assay that assessed the binding of soluble CD4 protein to purified gp120 from the JR-FL HIV-1 isolate revealed that **3** was a competitive and reversible inhibitor of protein association with a *K*_i_ value of 460 nM. This was a mechanism of HIV-1 inhibition that had been sought through the design of peptidomimetics based on the key recognition elements of CD4 but which had met with only limited success [16–18].

**Scheme 1:**
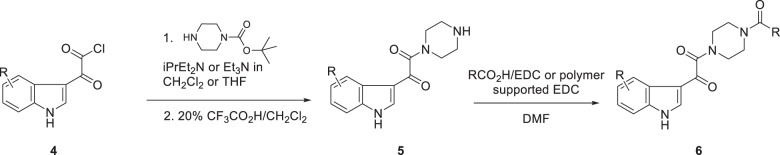
The synthetic approach utilized to explore variation of the phenyl moiety of the benzamide of **3**

As a lead molecule, **3** presented ample opportunity for structure-activity relationship (SAR) exploration, with variation of the benzoyl moiety the most straightforward survey that was pursued initially since piperazines **5** were readily prepared from **4** and acylated to afford **6**, as depicted in Scheme 1 [19]. Indole variation was studied in parallel, although this was a synthetically more challenging enterprise, with the 4-fluoro analog **15** the first to be prepared and evaluated (Table 1) [20]. Remarkably, this relatively simple molecular edit offered a significant 60-fold increase in antiviral activity that was a reliable SAR point based on the comparisons in Table 1; however, the origin of the enhancement in potency remains enigmatic but does not appear to be a function of the effect of the C-4 substituent on the conformation of the C-3 carbonyl moiety based on an analysis of single crystal X-ray structures. The SARs compiled in Table 1 are an informative selection of the compounds prepared and evaluated that highlight that the phenyl ring of the benzoyl moiety was poorly tolerant of structural variation, with even small substituents reducing antiviral potency [19, 20]. A survey of heterocycles as potential replacements for the phenyl ring revealed that only the lipophilic 2- and 3-thienyl analogs fully preserved potency and this early survey proved to be somewhat prescient since the benzoylated piperazine element of **3** is retained in **1** and **2** [7].

**Table 1:** Seminal SARs around the benzamide moiety in the context of the indole lead molecule **3** and the 4-fluoroindole core in the homolog **15**

	
Compd	R	EC_50_ (nM)	CC_50_ (μM)	Compd	R	EC_50_ (nM)	CC_50_ (μM)
**3**	C_6_H_5_	153	338	**15**	C_6_H_5_	2.6	>300
**7**	2-F-C_6_H_4_	391	101	**16**	2-thienyl	0.7	>300
**8**	3-F-C_6_H_4_	2,760	200	**17**	3-thienyl	0.4	>300
**9**	4-F-C_6_H_4_	8,900	280	**18**	4-thiazolyl	8.7	>250
**10**	2-pyridyl	1,550	>300	**19**	2-pyridyl	4.5	>300
**11**	3-pyridyl	4,200	>300	**20**	3-pyridyl	154	>300
**12**	C_6_H_5_CH_2_	>100,000	>300	**21**	4-pyridyl	24	>300
**13**	C_6_H_11_	3,500	>300	**22**	2-furanyl	5.4	>300
**14**	1-naphthyl	>100,000	>300	**23**	3-furanyl	20	>300

All antiviral data are generated from a JR-FL (M) *env* pseudotyped virus assay

**Scheme 2:**
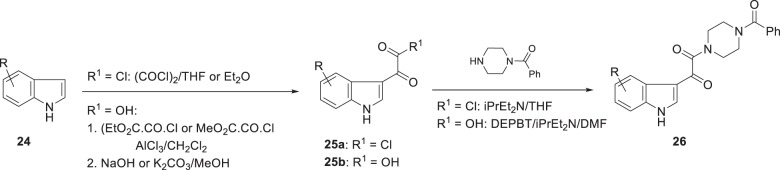
Methodology developed for the Friedel–Crafts acylation of substituted indoles with oxalyl chloride or a monoester of oxalyl chloride

**Fig. 1:**
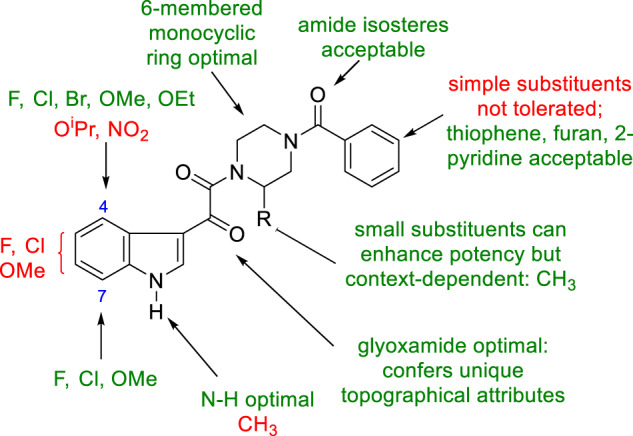
A synopsis of the key SAR points associated with structural variation of indole 3-glyoxamide-based HIV-1 AIs

The observation of the remarkable effect of the indole 4-fluoro substituent on potency provided a strong impetus to define the optimal heterocycle substitution pattern, which was explored extensively and systematically [7, 20]. In this series of analogs, the most straightforward synthesis relied upon acylation of a substituted indole **24** with oxalyl chloride and coupling of the acid chloride **25a** with *N*-benzoyl piperazine to afford the products **26**, as summarized in Scheme 2 [20]. Alternatively, acylation with methyl chlorooxalate or ethyl chlorooxalate in the presence of the Lewis acid AlCl_3_ afforded the glyoxyl ester product which, after saponification to the carboxylic acid **25b**, was annealed with *N*-benzoyl piperazine using 3-(diethoxyphosphoryloxy)-1,2,3-benzotriazin-4(3*H*)-one (DEPBT) as the coupling agent in the presence of Hünig’s base to provide target compounds **26** [20]. The seminal insights from this survey are synopsized in Table 2 where the effect of a F, Cl and OCH_3_ substituent at each of the four sites of the indole core provide a systematic matched SAR comparison that is illustrative of the tolerance to substitution at each of these sites of the pharmacophore. The broader SAR observations for this phase of the survey are summarized diagrammatically in Fig. 1. The data presented in Table 2 indicate that the halogens F and Cl and the small alkoxy substituents OMe and OEt at C-4 (**15**, **27**–**29**) and C-7 (**37**–**39**) are beneficial while the poor activity associated with the O^i^Pr substituent at C-4 in **30** is suggestive of a steric limitation at this site. In contrast, substitution at C-5 and C-6 with F, Cl or OMe was detrimental to HIV-1 inhibitory potency, with the effect slightly more severe at C-5 (**31**–**33**) compared to C-6 (**34**–**36**). The combination of C-4 and C-7 substituents further enhanced antiviral potency, as exemplified by **40**–**43**. However, a small but strongly electron withdrawing NO_2_ substituent at C-4 eroded potency as did alkylation of the indole N–H. Collectively, these SAR insights were to provide seminal guidance for the remainder of the discovery program.

**Table 2:** A synopsis of the SARs associated with indole substitution patterns in HIV-1 AIs


Compd	R^1^	R^2^	R^3^	R^4^	EC_50_ (nM)	CC_50_ (μM)
**15**	F	H	H	H	2.6	>300
**27**	Cl	H	H	H	4.3	212
**28**	CH_3_O	H	H	H	0.52	>300
**29**	CH_3_CH_2_O	H	H	H	0.45	>95
**30**	iPrO	H	H	H	>500	150
**31**	H	F	H	H	838.3	>300
**32**	H	Cl	H	H	395	46
**33**	H	CH_3_O	H	H	21,100	>300
**34**	H	H	F	H	21.1	>245
**35**	H	H	Cl	H	208	>110
**36**	H	H	CH_3_O	H	328.8	>300
**37**	H	H	H	F	7.3	>152
**38**	H	H	H	Cl	4.4	162
**39**	H	H	H	CH_3_O	6.6	153
**40**	F	H	H	F	0.35	>300
**41**	F	H	H	CH_3_O	0.06	>300
**42**	CH_3_O	H	H	CH_3_O	0.23	279
**43**	CH_3_O	H	H	Cl	0.07	>300

**Scheme 3:**
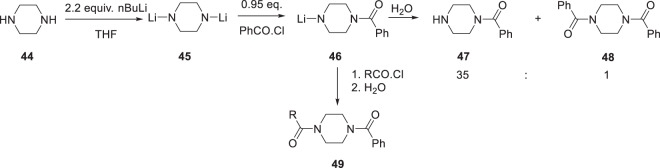
Mono-benzoylation of the lithium dianion derived from piperazine (**44**) and the synthesis of unsymmetrical piperazine diamides **49** [23]

**Scheme 4:**
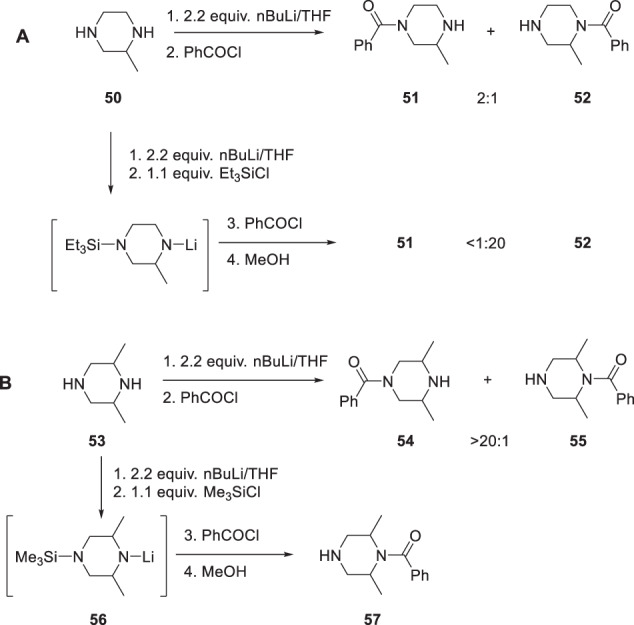
Synthetic protocols for the selective acylation of unsymmetrically-substituted piperazines [24]. **A** Synthetic protocol for the regioselective benzoylation of 2-methylpiperazine. **B** Synthetic protocol for the regioselective benzoylation of 2,6-dimethylpiperazine

**Scheme 5:**
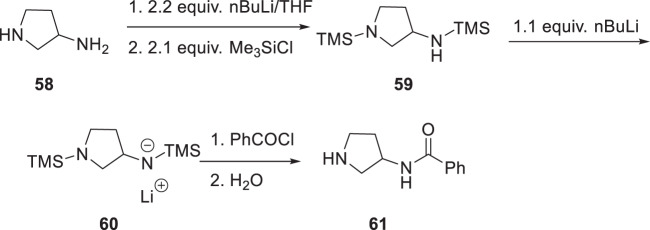
Synthetic protocol for the mono-benzoylation of pyrrolidin-3-amine (**58**) [25]

**Scheme 6:**
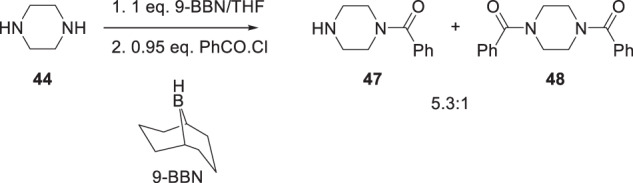
Synthetic protocol for the mono-benzoylation of piperazine (**44**) mediated by coordination of one amine moiety with 9-BBN [26]

A seam of new synthetic organic methodology was developed to facilitate a scan of piperazine substitution patterns in which methods to selectively mono-acylate a single nitrogen atom of symmetrically-substituted and unsymmetrically-substituted piperazines and other diamines were devised, as summarized in Schemes 3–6 [21–26]. The initial approach depicted in Scheme 3 took advantage of the kinetic reactivity of the piperazine dianion **45** prepared by exposure of **44** to 2.2 equivalents of n-butyllithium (nBuLi) in tetrahydrofuran (THF) [23]. The addition of 0.95 equivalents of benzoyl chloride provided the mono-benzoylated product **47** in 84% yield after an aqueous work-up, with only small amounts (~2%) of the bis-benzoylated material **48** formed (product ratio = 35:1). The intermediate mono-anionic **46** species could readily be captured by a second aroyl chloride to afford the unsymmetrically disubstituted compounds **49** in essentially quantitative yield. This methodology offered a practical solution to selective diamine modification that was successfully extended to homopiperazine [23].

For piperazines incorporating carbon-based substituents deployed in an unsymmetrical topology, control over the regioselectivity of acylation was accomplished by taking advantage of steric effects and the temporary in situ protection of one of the nitrogen atoms, as summarized in Scheme 4 [24]. For mono-alkylated piperazines like 2-methylpiperazine (**50**), the regioselectivity of acylation was modest (Scheme 4A). Thus, treatment of **50** with 2.2 equivalents of nBuLi followed by the addition of benzoyl chloride afforded a 2:1 mixture of the mono-benzoylated products **51** and **52**, with the less sterically hindered product favored. However, by trapping the dianion of **50** with 1.1 equivalents of Et_3_SiCl and then adding benzoyl chloride, the regioselectivity could be reversed, with **52** the dominant product by a ratio that was in excess of 20:1. The more sterically demanding 2,6-dimethylpiperazine (**56**) offered a much higher degree of regioselective control in this process, with the mono-benzoylated product **54** isolated in 80% yield accompanied by only small amounts of the more sterically congested isomer **55** (ratio = >20:1) (Scheme 5B). However, the regioselectivity ratio for this reaction could also be reversed by temporarily protecting the less hindered amine of **53** in situ, with experimentation revealing the Me_3_Si moiety to be the optimal solution (Scheme 4B). Thus, the dianion derived from **53** was treated with 1.1 equivalents of Me_3_SiCl for 1 h at room temperature to give an intermediate mono-anion **56** to which was added 0.95 equivalents of benzoyl chloride and the mixture stirred for 10 min before being quenched with MeOH. In the case of 2,6-dimethylpiperazine, the more sterically encumbered product **57** was isolated in 99% yield after chromatography. This in situ protection process was extended to the regioselective acylation of primary amines in the presence of secondary amines, as illustrated in Scheme 5 where the process is demonstrated with pyrrolidin-3-amine (**58**) as the substrate. In this experimental protocol, treatment of **58** with 2.2 equivalents of nBuLi in THF at room temperature afforded a dianion with was doubly protected in situ as the bis-trimethylsilyl derivative **59** by the addition of 2.1 equivalents of Me_3_SiCl. After stirring for 30 min, 1.1 equivalents of nBuLi were added to generate the mono-anion **60** which was followed 30 min later by 0.95 equivalents of benzoyl chloride to afford **61** in 95% yield after an aqueous work-up and chromatographic purification [24].

An alternative and complementary approach to the mono-acylation of diamines was also developed that relied upon the selective pre-complexation of a single nitrogen atom of a primary or secondary diamine with the Lewis acidic 9-borabicyclo[3.3.1]nonane (9-BBN), the best of 38 reagents screened [26]. For piperazine (**44**), stirring a solution of the heterocycle in dry THF with 1 equivalent of 9-BBN in hexane for 1 h at room temperature followed by the addition of 0.95 equivalents of benzoyl chloride and stirring for an additional 1 h afforded a mixture of the mono-benzoylated **47** and bis-benzoylated **48** products in 84% combined yield in a ratio of 5.3:1 (Scheme 6). This process was successfully expanded to mono-benzoylate a range of acyclic and cyclic diamines that included ethylenediamine, *N,N’*-dimethyl-1,2-ethylenediamine, higher congeners and homopiperazine, all of which were examined for their potential to replace the piperazine moiety of HIV-1 AIs [26].

The compounds compiled in Table 3 provide a synopsis of the results of the survey of piperazine substitution and replacements that were explored in the context of the 4-fluoroindole core [21]. Mono-methylation proximal to the glyoxamide moiety led to a 5-fold enhancement of antiviral potency in the context of **62**, with the (*R*)-isomer **63** ~10-fold more potent than the (*S*)-antipode **64**. Installing a single methyl substituent proximal to the benzamide afforded an analog **65** that was comparable to the progenitor **15** while larger alkyl substituents at both sites led to reduced antiviral potency. Piperazine ring expansion (**66**), steric constraint with a bridging element (**67**), ring opening (**68**–**71**), deployment on a cyclohexane scaffold in either a 1,2- (**72** and **73**) or 1,3-topology (**74**) resulted in poorly active compounds, with only the *cis*-diamino cyclohexane **73** exhibiting detectable antiviral activity in the pseudotyped virus assay at a concentration of less than 300 μM.

**Table 3:** A synopsis of the effect of piperazine substitution or replacement on antiviral activity of 4-fluoro-substituted indole 3-glyoxamide derivatives [21]


Compd #		EC_50_ (nM)	CC_50_ (μM)
**15**		2.6	>300
**62**		0.56	>300
**63**		<0.16	>300
**64**		1.38	>300
**65**		2.60	>300
**66**		>50,000	>300
**67**		>50,000	>300
**68**	NH-CH_2_-CH_2_NH	>50,000	>300
**69**	CH_3_N-CH_2_-CH_2_N-CH_3_	>50,000	>300
**70**	NH-CH_2_-CH_2_-CH_2_NH	>50,000	>300
**71**	CH_3_N-CH_2_-CH_2_-CH_2_-NCH_3_	>50,000	>300
**72**		>50,000	>300
**73**		30,500	65
**74**		>50,000	>300

**Scheme 7:**
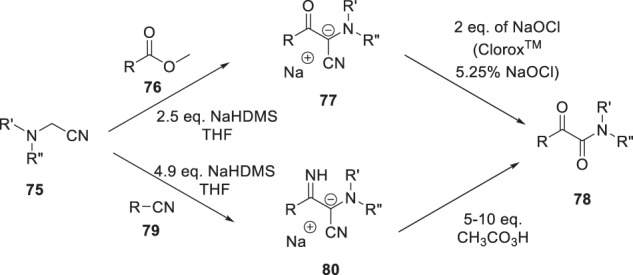
A synthetic protocol to access glyoxamides **78** by a Claisen-type condensation between an aminoacetonitrile **75** and an ester **76** followed by oxidation of the intermediate anion **77** or by the base-mediated coupling of **75** with a nitrile **79** to afford an intermediate anion **80** which was converted to **78** by an oxidative process [27, 28]

The paucity of commercially available reagents that would facilitate a survey of indole glyoxamide derivatives and the challenge presented by synthesizing that structural element of HIV-1 AIs resulted in the invention of several methodologies that had wider ranging applications in organic synthesis. These synthetic methodologies revolved around the reactivity of the anions of aminoacetonitrile derivatives that facilitated an extensive evaluation of the effect of structural variation of the core indole heterocycle [27, 28]. An additional element that was given consideration in the development of this methodology was the identification and optimization of chemistries that were effective at ambient temperatures and over extended reaction times designed to maximize convenience and applicability. This was a practical aspect that facilitated these surveys by taking advantage of reactions conducted overnight or in batches in parallel with the more pressing imperatives in the program which were pursued as the primary focus. In the initial iteration of this methodology which is detailed in Scheme 7, a Claisen-type condensation between an aminoacetonitrile derivative **75** and an ester **76** mediated by a 2.5-fold excess of sodium bis(trimethylsilyl)amide (NaHDMS) afforded an intermediate anion **77** that is stabilized by both the electron withdrawing ketone and nitrile substituents [27]. This intermediate was oxidized in situ to afford an unstable cyanohydrin that collapsed with the loss of cyanide to generate the α-keto amide product **78**. Investigation of a series of oxidizing agents identified aqueous NaOCl as optimal, a convenient reagent that is commercially available as the bleach Clorox^TM^, a 5.25% solution of NaOCl in H_2_O. Alternatively, coupling of the aminoacetonitrile derivative **75** with a nitrile derivative **79** in the presence of 4.9 equivalents of NaHDMS afforded an imine intermediate **80** which was oxidized to **78** using, in this case, a 5- to 10-fold excess of CH_3_CO_3_H [27].

**Scheme 8:**
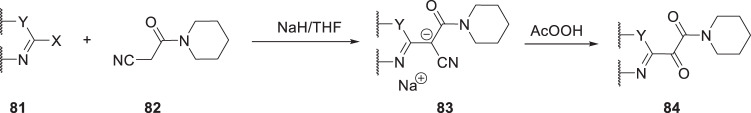
A synthetic protocol developed to access heterocycle-based α-keto amides **84** [29]

A variant on this process that is depicted in Scheme 8 allowed access to derivatives of **78** that incorporated a heterocycle [29]. An S_N_Ar substitution of a 2-halo heterocycle **81** with the anion of 1-cyanoacetylpiperidine (**82**) afforded the stabilized anion **83** which, upon exposure to CH_3_CO_3_H, generated an unstable cyanohydrin that collapsed to the α-keto amide **84**.

The base-mediated S_N_Ar reaction of a 2-halo heterocycle **81** with an α-aminoacetonitrile (**75**) or a (hetero)aryl acetonitrile (**85**) as the coupling partner afforded the intermediate stabilized anions **86** and **87**, which provided **88** and **89** after exposure to an oxidant [30, 31]. For the anion **86**, the optimal oxidants were NiO_2_ or bis(trimethylsilyl)peroxide, although yields exhibited some dependency on the identity of the heteroaryl halide, while for **87**, sodium peroxide was the preferred oxidizing reagent.

As summarized in Scheme 10, when malononitile (**90**) was used in this reaction manifold, oxidation of the intermediate anion **91** with CH_3_CO_3_H afforded a cyanohydrin derivative that collapsed to the acyl nitrile **92**, an electrophilic species that readily reacted with amines to afford α-keto amides **88** [32]. From a practical perspective, the amine was added to the solution of **91** just prior to the introduction of CH_3_CO_3_H, a protocol that constituted a convenient, one-pot process to deliver amides **88** in yields ranging from 29 to 67%.

**Scheme 9:**
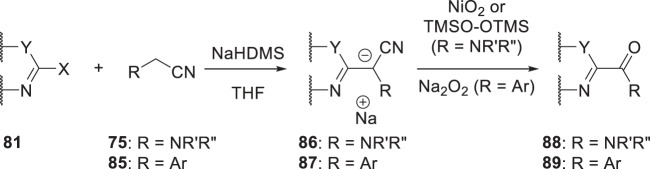
Synthetic protocol to access amides **88** and ketones **89** by reaction of an aminoacetonitrile **75** or a heteroarylmethyl nitrile **85** with a 2-halo heterocycle **81** followed by oxidation of the intermediate anions **86** and **87** [30, 31]

**Scheme 10:**
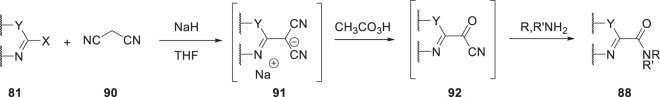
A synthetic approach to the preparation of heterocycle-containing amide derivatives by sequential reaction a 2-halo heterocycle with malononitrile (**90**), CH_3_CO_3_H and an amine to afford amides **88** via the intermediacy of an acyl nitrile **92** [32]

**Scheme 11:**
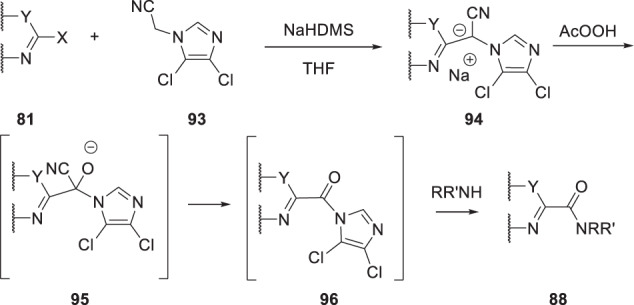
A synthetic approach developed to access heteroaryl carboxamides **88** [33]

A variation on this methodology hybridized elements of the reactions described in Schemes 8, 9 and 10 with the unique properties associated with azole-*N*-acetonitriles to generate an acylimidazole intermediate that has reactivity analogous to the acyl nitrile **92** [33]. As summarized in Schemes 11, 2,3-dichloroimidazole proved to be the optimal azole heterocycle in the context of **93** which was subjected to a base-mediated coupling with a 2-halo-substituted heterocycle **81** to ultimately afford amides **88**. Thus, a mixture of **81** and **93** was stirred in the presence of an excess of NaHDMS for 10 h to generate the intermediate anion **94** to which was added an excess of amine followed by CH_3_CO_3_H and the mixture stirred for another 10 h to deliver the products **88** in yields ranging from 31 to 87%. This process relies upon oxidation of the anionic intermediate **94** to give the cyanohydrin **95**, which collapses to the acyl imidazole intermediate **96**, thereby setting the stage for reaction with an amine to give amides **88** [33].

**Scheme 12:**
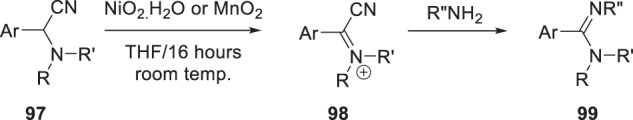
Reaction protocol for the oxidation of aminoacetonitrile derivatives **97** and capture of intermediate **98** to afford amidines **99** [34]

While cyanohydrin derivatives were proposed as intermediates for reaction processes that involved anionic species, consideration of a pathway involving the formation of an iminium cation **98** from an α-aminoacetonitrile **97** suggested the potential to generate amidines **99** using this chemistry, as delineated in Scheme 12 [34]. In this protocol, **97** was stirred with an excess of NiO_2_.H_2_O or MnO_2_ for 16 h followed by the addition of an amine to produce amidines **99** in yields that ranged from 8 to 97%. However, some customization of the conditions was required, with higher yields typically observed with the more nucleophilic amine partners although electron deficient and poorly nucleophilic amine derivatives also participated. The lowest yield was observed with the weakly nucleophilic *N,N*-dimethyl sulfamide as the amine component although NH_2_CN afforded products in 51–64% yield [34].

**Fig. 2:**
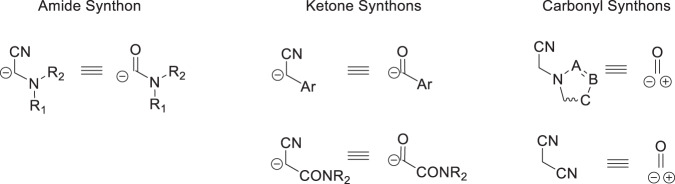
Functional equivalences of the synthons developed to access HIV-1 AIs

A summary of these methodologies and the functional equivalencies of the reagents developed as amide, ketone and carbonyl synthons is depicted schematically in Fig. 2.

These synthetic methodologies were used to conduct a broad-ranging survey of potential indole replacements that encompassed almost 30 different heterocycles and carbocycles, depicted synoptically by **100**–**112** which were some of the most promising inhibitors to emerge from this study, with the key antiviral data compiled in Table 4 [28]. While this survey surfaced several interesting SAR observations that were largely explained by considering simple two-dimensional structural overlays with substituted indole derivatives, none of the compounds offered sufficient inherent advantage to be given further consideration. Perhaps not surprisingly given the marked structural variation explored, the SARs diverged somewhat from that of the indole-based prototypes. For example, the *N*-methyl benzimidazole-based inhibitor **102** is 60-fold more potent than the unsubstituted analog **101**, an SAR point that contrasts with the indole series where the N–H is a critical element. However, in this series, the piperazine methyl substituent in **101** confers a similar 3-fold advantage over **100** that is consistent with observations made in the indole series. The antiviral activity associated with the other examples compiled in Table 4 confirm that in these combinations of topology and core element, the presence of an N–H is not a prerequisite for potent inhibition of HIV-1 replication. However, antiviral activity is sensitive to the identity of the indole replacement (for example, compare benzothiazole **103** with oxazole **104**), with many of the more active analogs presenting planar, lipophilic ring systems.

**Table 4:** A synopsis of the effect of replacements for the indole heterocycle in HIV-1 AIs [28]


	Ar	R	EC_50_ (nM)	CC_50_ (μM
**100**		H	2,460	>300
**101**		(*R*)-CH_3_	798	>300
**102**		(*R*)-CH_3_	12	>300
**103**		(*R*)-CH_3_	34	>230
**104**		H	>5,000	>300
**105**		H	135	94
**106**		(*R*)-CH_3_	2.4	171
**107**		H	355	>300
**108**		H	13	>300
**109**		CH_3_	1.2	266
**110**		(*R*)-CH_3_	0.7	198
**111**		(*S*)-CH_3_	4.4	166
**112**		H	182	>300

The antiviral profile of the 4-fluoro analog **15** was examined across a panel of HIV-1 isolates where it performed encouragingly well and the compound was advanced into pharmacokinetic (PK) studies as a prototype molecule with which to identify potential liabilities associated with the chemotype [6]. The bioavailability of **15** in dogs and cynomolgus monkeys following oral dosing as a solution in a 90:10 v/v mixture of poly(ethylene glycol) 400 (PEG 400) and ethanol was complete but much lower in the rat (9.4%), a not unanticipated observation given the poor metabolic stability of **15** in rat liver microsomes (RLMs). However, dose escalation studies in rats revealed non-linear plasma exposure, suggestive of solubility and/or dissolution issues which were brought into clear focus when **15** was dosed to rats and dogs as a suspension formulation at a particle size of 46 μm where the oral bioavailability fell to 4% and 36%, respectively [6].

**Scheme 13:**
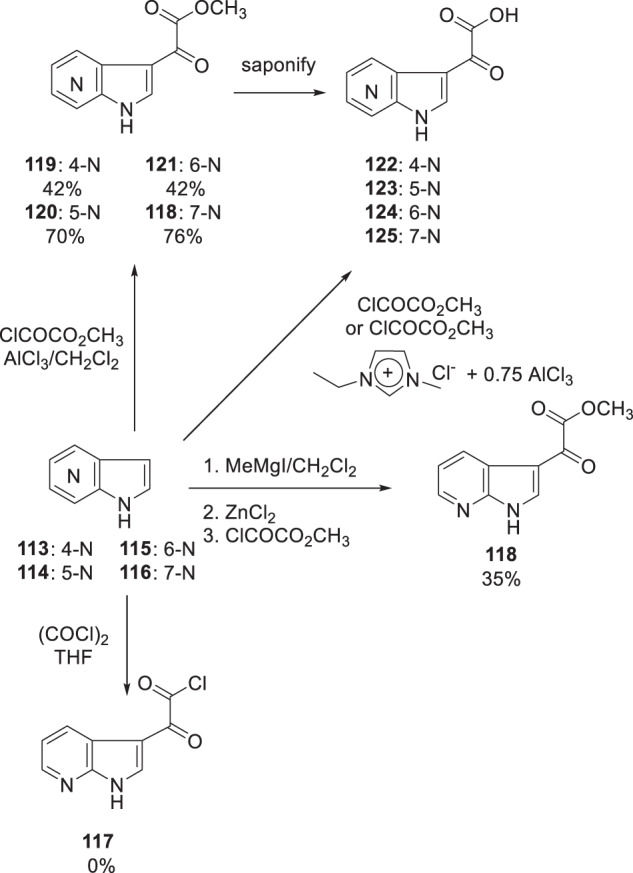
Methodologies devised to provide synthetic access to azaindole 3-glyoxylic acid derivatives [35, 40, 41]

As a consequence of this observation, further study of **15** was abandoned and attention was focused on addressing the pharmaceutics issues with new molecules. To this end, a systematic scan of the effect of introducing a nitrogen atom into the indole core was examined in the context of the preferred methylated piperazine moiety. This provided a series of four azaindoles and an indazole analog that were designed to enhance intrinsic heterocycle polarity with the anticipation of a positive effect on the physicochemical properties of the molecule [35–38]. However, prosecuting this phase of the project presented synthetic challenges associated with access to the parent azaindole structures as well as their elaboration by substitution at the C-3 position. For the latter problem, as electron-deficient heterocycles, acylation at the C-3 position of azaindoles under conditions that were effective for indoles was neither straightforward nor reliable, as exemplified by the absence of product **117** when 7-azaindole (**111**) was reacted with oxalyl chloride which contrasted with the high yield of the indole adduct, as summarized in Scheme 13 [39]. This provided a clear practical problem that stimulated the development of reaction protocols that would deliver the glyoxylated products reliably and in reasonable and practical yields. The initial solution devised to access the 7-azaindole glyoxalate ester **118** relied upon increasing the nucleophilicity at C-3 by deprotonating the heterocycle N–H with MeMgI, adding ZnCl_2_ followed by methyl oxalyl chloride, which delivered **118** in a modest but practically useful 35% yield given the convenience of the protocol [6]. However, a more generally applicable solution that was devised took advantage of the inclusion of the Lewis acid AlCl_3_ to catalyze the reaction of azaindoles **113**–**116** with methyl oxalyl chloride in CH_2_Cl_2_ as the solvent, as depicted in Scheme 13 [35, 39]. This procedure provided the 4-aza (**119**), the 5-aza (**120**), the 6-aza (**121**) and 7-aza (**118**) glyoxalate esters in yields of 42%, 70%, 42% and 76%, respectively. The ester moieties were readily saponified to the acids **122**–**125** which were coupled with the 3-methyl-substituted benzoylated piperazine to afford the targeted compounds [35]. An alternative process developed for the acylation of electron-deficient indoles and 6-azaindoles utilized the highly acidic ionic liquid 1-ethyl-3-methylimidazolium chloroaluminate, generated from 1-ethyl-3-methylimidazolium chloride (EmimCl) and aluminum chloride (0.75(AlCl_3_), as the solvent and catalyst, respectively [40, 41]. Stirring a mixture of indole or azaindole with methyl chlorooxalate or ethyl chlorooxalate at room temperature for 18 h in the ionic liquid effected acylation of substituted 4- and 6-azaindoles followed by ester dealkylation to deliver substituted variants of the acids **122** and **124** in yields ranging from 43 to 90% [40, 41].

**Scheme 14:**
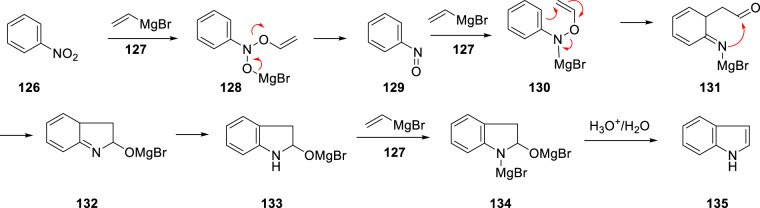
Mechanism of the Bartoli indole synthesis of indole (**135**) from nitrobenzene (**126**) [42, 43]

Synthetic approaches to access the azaindoles were also required in order to more fully enable this aspect of the SAR survey. Surprisingly, the Bartoli indole synthesis, a convenient, one-pot although somewhat low yielding process, had not been explored with nitropyridine-based substrates. This reaction proceeds by the complex mechanism illustrated in Scheme 14 for the reaction of nitrobenzene (**126**) with an excess of vinylmagnesium bromide (**127**) to produce indole (**125**) [42, 43]. The mechanism that has been deduced as the result of careful experimentation involves attack of **127** on one of the oxygen atoms of the nitro moiety of **126** to generate the magnesium salt of *N*-hydroxy-*N*-phenyl-*O*-vinylhydroxylamine (**128**) which readily collapses to nitrosobenzene (**129**). A second equivalent of **127** converts **129** to **120** which sets the stage for a 3,3-sigmatropic rearrangement to generate the aldehyde **131**, an intermediate that is poised to ring close to form the heterocyclic core **132**. Rearomatization to **122** by tautomerization followed by deprotonation of the N–H by a third equivalent of **127** affords **134** which after quench with aqueous acid eliminates H_2_O to generate **135** [42, 43]

**Scheme 15:**
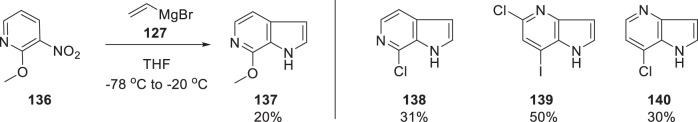
Preparation of 4- and 6-azaindoles from nitropyridine derivatives using the Bartoli indole synthesis protocol [45, 46]

The Bartoli protocol was successfully extended to the preparation of 4- and 6-azaindoles from the respective nitro-substituted pyridines, as summarized in Scheme 15 [44–46]. A prototype reaction was conducted on 2-methoxy-3-nitropyridine (**136**) which was exposed to a 3-fold excess of **127** in THF at −78 °C before warming the mixture to −20 °C and stirring for 8 h to afford the 6-azaindole **137** in 20% yield after chromatographic purification. Although the yield of **137** was modest, it was comparable to those typically obtained in the preparation of indoles using this protocol and offered convenience based on the availability of starting materials. Moreover, although the yields were low on typical lab scales, the reactions were clean based on chromatographic analysis, with the products usually isolated straightforwardly by flash chromatography which primarily separated baseline materials from the targeted azaindole. Two phenomena were noted for this procedure: higher yields were obtained when larger substituents were incorporated adjacent to the NO_2_ group or when there was a halogen substituent at the 2- or 4-position with respect to the NO_2_ moiety, as exemplified by **138**–**140**. The latter compounds were of particular value because these substituents provided useful functionality that allowed further synthetic manipulation. For **138**, which was isolated in 31% yield, the parent 6-azaindole **115** was readily obtained in 86% yield by a simple hydrogenolysis reaction [45].

**Scheme 16:**
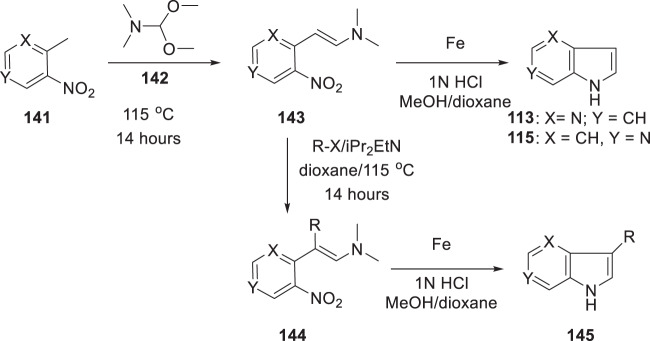
An approach to the synthesis of azaindoles based on the Leimgruber–Batcho reaction protocol [49]

A second approach to access azaindoles relied upon the two-step Leimgruber–Batcho reaction protocol which was known to deliver 4- and 6-azaindoles [44, 47, 48]. In this process, a 2-methyl-substituted nitropyridine **141** was dissolved in DMF-dimethyl acetal (**142**) and the mixture heated at 115 °C for 14 h to afford the enamine **143** (Scheme 16) [49]. Reduction of the nitro moiety using Fe in a mixture of 1N HCl, MeOH and dioxane furnished the azaindole **113** or **115** after cyclization with concomitant elimination of dimethylamine. However, the synthetic versatility of this process was extended by alkylating the enamine intermediate **143** with alkyl halides or a Michael acceptor to provide **144** which afforded 3-substitued 4- or 6-azaindoles **145** after reduction of the nitro moiety and cyclization.

**Fig. 3:**
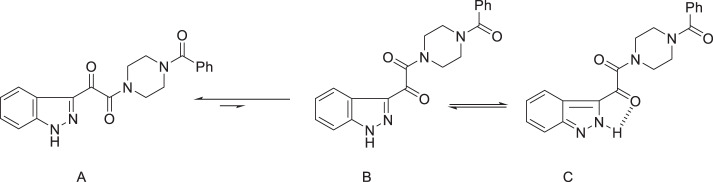
Conformational flexibility and tautomerism of the indazole-based HIV AI 151 [28, 51]. **A** Preferred orientation of the 3-carbonyl moiety; **B** alternative orientation of the 3-carbonyl moiety; **C** intramolecular H-bonded tautomer of B

An examination of the properties of the azaindole derivatives that are compiled in Table 5 proved to be both insightful and instructive [35, 36]. The 4- and 7-azaindoles **147** and **150**, respectively, fully retained the antiviral potency of the progenitor indole **146** while the 6-aza analog **149** was 5-fold weaker and the 5-aza derivative **148** was substantially less active and the poorest performer in this cohort [35]. The effects of aza substitution introduced a weakly basic site for which the p*K*_a_ values ranged from 2.0 for **140** to 6.2 for **148** while the effect on the p*K*_a_ value of the N–H was more consistent, with an approximate one unit reduction across the series compared to **146**. All four isomers **147**–**150** exhibited lower Log*D*_6.5_ values than the prototype **146**, reflective of an increase in polar surface area, although the correlation with cLog*P* and cLog*D*_7.0_ values is poor. The increased polarity translated into improved aqueous solubility, enhanced by 25-fold for **148** and **149** and 58-fold for **147** and **150** compared to the indole **146**, although there was a poor correlation with the melting point. However, the increase in polarity with azaindoles compromised membrane permeability in a Caco-2 cell assay, an effect that was more severe for the 5-aza and 6-aza analogs **148** and **149**, respectively, where the nitrogen atom is more exposed. Stability in human liver microsomes (HLMs) was improved for all four isomers and concerns about the potential for introducing potentially problematic CYP 450 3A4 inhibition were allayed by experimental evaluation where only the 5-aza analog **148** was found to weakly inhibit the metabolism of two known substrates [35]. The final permutation of nitrogen scanning that was explored was with the indazole **151** which was poorly active, comparable to **148**, an antiviral profile that disfavored this heterocyclic core as a potential vehicle for further study [28]. The poor activity of **151** was attributed to the effect of the introduction of the ring nitrogen atom on the conformational mobility around the indazole C-3 junction, as summarized in Fig. 3, which favors form A. In form B, there is the potential for an intramolecular H-bond to the C-3 carbonyl oxygen atom (form C), all of which presumably restricts access to the conformational form preferentially recognized by HIV-1 gp120 [28, 50, 51].

**Table 5:** The effect of introduction of a nitrogen atom into the indole core of HIV-1 AIs on antiviral activity, physicochemical properties and select developability attributes [35]


Compound #	146	147	148	149	150
Property	indole	4-*N*	5-*N*	6-*N*	7-N
EC_50_ (nM)	4.0	1.6	576	21.6	1.7
CC_50_ (μM)	200	>300	>300	>300	280
Log*D*_6.5_	1.9	0.9	1.2	1.5	1.8
cLog*P*/cLog*D*_7.0_	2.70/2.70	1.92/1.92	1.36/1.32	2.36/2.30	1.79/1.79
PSA (Å)^2^	73.5	86.4	86.4	86.4	86.4
Cryst. sol. at 25 °C, pH 6.5 (mg/mL)	0.016	0.93	0.42	0.49	0.94
mp (^o^C)	212–216	298–301	227	249–253	146–148
p*K*_a_	10.9	5.0/9.8	6.2/9.8	6.0/9.3	2.0/9.7
Caco-2 Pc (nm/sec)	169	76	19	<15	168
HLM t_½_ (min)	16.9	>100	>100	38.5	49.5
CYP 450 3A4 inhibition IC_50_ (μM)	>40/>40	>40/>40	18/9.3	>40/>40	>40/>40
hERG inhibition IC_50_ (μM)	>80	>80	>80	>80	>80

cLog*P*/cLog*D*_7.0_ and PSA values are calculated using Advanced Chemistry Development (ACD/Labs) Software V11.02 (^©^1994-2021 ACD/Labs) with the data abstracted from SciFinder®

CYP 3A4 inhibition was assessed using the 2 substrates benzyloxy-4-(trifluoromethyl)coumarin and 7-benzyloxyresorufin.


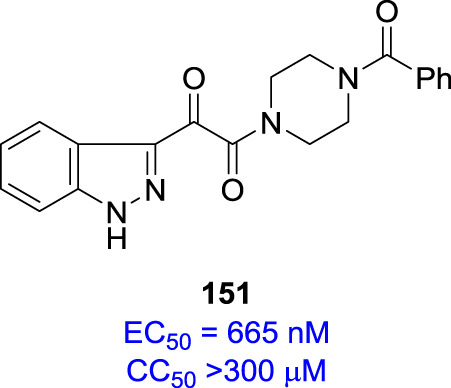

**Fig. 4:**
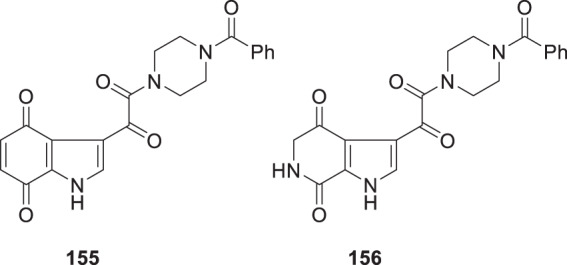
Structures of **155** and **156**, potential metabolites of **42** and **154**, respectively

While the poor intrinsic antiviral potency of the 5-azaindole analog **148** eliminated this heterocycle topology from further consideration, the other three isomers offered potential for further study since it was anticipated that the physicochemical and antiviral properties of these molecules could be further refined by the introduction of substituents. The 4-azaindole series was studied in some detail and although several potent compounds that exhibited good PK properties were identified, these were ultimately set aside in favor of the C-6 and C-7 azaindole isomers, a fate that also befell the 4,6-diaza chemotype **152** [46, 52]. The overall developability properties of the 7-azaindole core in **150** were attractive and the 4-methoxy substituted derivative **153** (BMS-378806) offered an antiviral profile that allowed its selection as the first candidate to be advanced into clinical study [6]. Unfortunately, the plasma exposure of **153** in a single ascending dose (SAD) study conducted in normal healthy volunteers (NHVs) failed to achieve a concentration that was above the targeted protein-binding adjusted EC_90_ value and the compound was abandoned. By this time in the program, attention had become firmly focused on the 6-azaindole series **149** since this heterocycle offered the combination of reasonable inherent antiviral potency with the unique potential within the azaindole series to substitute at the key C-4 and C-7 sites of the core scaffold where antiviral potency could be optimized based on studies conducted with the indole series. The 4,7-dimethoxy substituted derivative BMS-488043 (**154**) emerged as the second candidate to be advanced into clinical trials, a compound of significance to the program since it demonstrated clinical proof-of-concept for this mechanistic approach to controlling HIV-1 infection [35]. BMS-488043 (**154**) is the 6-aza analog of the 4,7-dimethoxy-substituted indole **42** that had piqued interest based on its intrinsic antiviral potency but the introduction of the nitrogen atom abrogated the potential formation of the quinone **155**. Quinone **155** was considered to be a possible metabolite of **42** since *O*-demethylation was observed in liver microsomal studies in vitro, giving cause for concern based on the known electrophilicity of this heterocyclic class (Fig. 4) [53, 54]. In contrast, complete demethylative metabolism of **154** would produce the amide **156**, a comparatively benign compound since it does not present a substituted electrophilic quinone moiety.
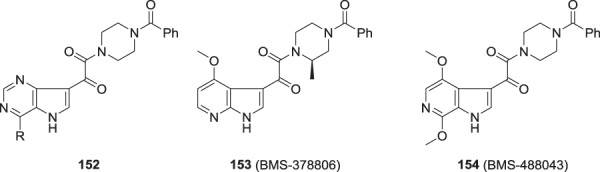

**Fig. 5:**
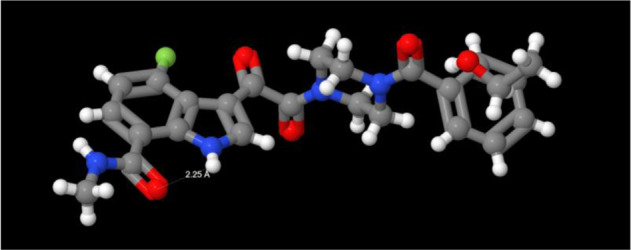
Single crystal X-ray structure of mono-methyl amide **158** illustrating the intramolecular H-bond between the amide C=O and indole N–H

**Fig. 6:**
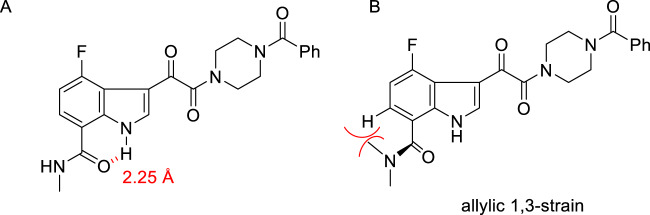
**A** Intramolecular H-bond between the amide C=O and indole N–H in **158**; **B** Allylic-1,3-type strain between the trans-methyl substituent of the dimethylamide of **159** and the C-6 proton of the indole core

While **154** was navigating pre-clinical toxicology studies along the regulatory path to filing an investigational new drug (IND) application, developing a deeper understanding of HIV-1 AI pharmacophore continued, with optimization heavily focused on elaboration of the C-7 position which was explored extensively in the context of both indole and 4- and 6-azaindole cores [46, 52, 55, 56]. Modifications at this site were the subject of extensive study, with many avenues pursued simultaneously that explored a broad range of functionalities, with a significant emphasis on heterocycles that were both *C-* and *N*-linked in their connectivity to the core. The challenges were to combine potent antiviral activity with targeted PK properties, with latter more typically associated with *N*-linked azole heterocycles which ultimately emerged as the preferred chemotype. The broad seam of SAR observations developed during this phase of the program coalesced around the notion that coplanarity between the core heterocycle and the C-7 substituent, which was either an amide or an azole or azine heterocycle, was important for antiviral potency [46, 52, 55, 56]. An effective encapsulation of this phenomenon can be seen in the SARs associated with the three indole-7-carboxamide derivatives **157**–**159** that are compiled in Table 6 [55]. The HIV-1 inhibitory potency of the primary amide **157** was similar to that of the progenitor **15** and was enhanced 4-fold by the mono-methyl homolog **158**. However, the dimethylamide derivative **159** was a considerably less potent HIV-1 inhibitor, with an over 800-fold reduction in activity compared to **158**. A single crystal X-ray structure of **158** revealed that the amide carbonyl engaged the proximal indole N–H in an intramolecular H-bonding interaction that conferred planarity to this region of the molecule, essentially conferring a pseudo ring system (Figs. 5 and 6A) [55, 56]. While the primary amide **157** would be able to adopt a similar conformation, the introduction of the second methyl substituent in **160** would introduce an unfavorable steric interaction between the H-atom at C-6 of the indole core and the amide N-CH_3_ substituent that is disposed *trans* to the C=O. This is a form of allylic 1,3-strain that would lead to a distortion of the amide moiety from planarity despite the presence of an intramolecular H-bond between the amide C=O and indole N–H, as depicted in Fig. 6B [57–61]. Interestingly, the 4-fold difference in potency between **157** and **158** was not reproduced in the 4-methoxy series or when the preferred methylated piperazine moiety was incorporated where potency was actually 5-fold weaker for this specific amide edit. The somewhat mercurial nature of the SAR in this region of the HIV-1 AI pharmacophore was further underscored by studies of homologation of the amide moiety of **158**, a survey that provided additional insight into both the size of the substituent tolerated at C-7 and the physical properties that were compatible with potent antiviral activity. The sampling of compounds prepared and evaluated that are compiled in Table 6 reveal interesting SAR points, with the ether **160** and amine **161** offering similar potency to each other but both are 25-fold weaker than progenitor **158** [55]. Double methylation of **161** led to a further 25-fold erosion of antiviral potency (**162**). A significant difference in potency was also observed between the homologous benzimidazole derivatives **163** and **164**, with the 2-amino compound **164** a picomolar HIV-1 inhibitor that was 160-fold more potent than homolog **163**. Replacing the benzimidazole of **164** with the more lipophilic benzothiazole heterocycle found in **165** reduced potency by 8-fold; however, the truncated thiazoles **166** and **167** were the most impressive compounds to emerge from this series with EC_50_ values of approximately 6 pM in the pseudotyped virus assay. Restoration of an aromatic element in the thiazole series gave **168**, in essence a deannelated analog of **165**, that was comparable to the fused ring prototype. However, despite an extensive study of indole amides, identifying a compound in this series that combined antiviral potency with targeted ADME properties proved to be elusive, with metabolic stability and membrane permeability the key liabilities. None of the analogs prepared as part of this survey improved on the profile of the methyl amide **158**, the most promising member of this series, which offered targeted PK properties but which suffered from an unacceptably large serum effect in the in vitro antiviral assays, preventing further consideration of this compound as a development candidate [55].

**Table 6:** SARs associated with HIV-1 AIs substituted at C-7 with a carboxamide moiety


	R	R’	EC_50_ (nM)	CC_50_ (nM)
**157**	H	H	2.03	>300
**158**	H	CH_3_	0.52	>300
**159**	CH_3_	CH_3_	407	>300
**160**	H	CH_2_CH_2_OCH_3_	18.8	298
**161**	H	CH_2_CH_2_NH_2_	12.8	135
**162**	H	CH_2_CH_2_N(CH_3_)_2_	315.7	>300
**163**	H		6.7	>300
**164**	H		0.04	9.2
**165**			0.32	81
**166**			0.0057	89
**167**			0.0058	29
**168**			0.21	>300

**Fig. 7:**
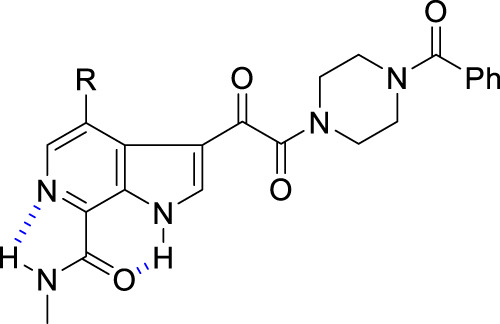
Intramolecular H-bonding interactions in C-7 mono-methyl carboxamide-substituted 6-azaindoles

The installation of a C-7 monomethyl amide substituent in the C-6 azaindole series provided additional insights that favored further pursuit of this core heterocycle chemotype [62]. As delineated in Table 7, **169**, the 6-aza analog of **158**, provided a 5-fold boost in antiviral potency that was accompanied by a modest increase in membrane permeability and metabolic stability but a more significant reduction in protein binding [62]. Comparison of the more polar 4-methoxy matched pairs **170** and **171** confirmed these observations, with the free fraction of **171** significantly enhanced while the presence of the 6-aza heterocycle also led to improved membrane permeability in this comparison, attributed to a reduction in solvation of the amide N–H by the formation of an intramolecular H-bond, as depicted in Fig. 7 [63, 64].

**Table 7:** SARs and in vitro profiling of indole and 6-azaindole C-7 carboxamide derivatives


	X	R	EC_50_ (nM)	CC_50_ (μM)	Caco-2 (nm/s)	HLM t_1/2_ (min)	Human plasma protein binding
**158**	C	F	0.52	>300	112	81	99%
**169**	N	F	0.09	>300	124	>100	96%
**170**	C	OCH_3_	0.76	>300	39	40	N.D.
**171**	N	OCH_3_	0.28	>150	132	100% rem.	76%

**Fig. 8:**
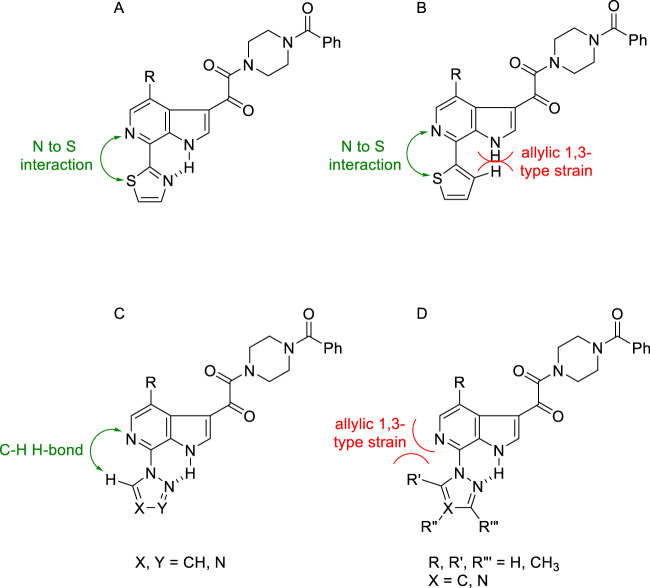
Favorable and unfavorable interactions between C-7 heterocycles and the 6-azaindole core in a series of HIV-1 AIs that stabilize or destabilize a planar topography. **A** Stabilization of a thiazole ring conformation by a favorable nitrogen to sulfur σ* and a thiazole nitrogen to azaindole N-H H-bond interaction; **B** Thiophene conformation stabilization by a favorable nitrogen to sulfur σ* interaction offset by unfavorable allylic 1,3-type strain between the thienyl 3-hydrogen atom and the azaindole N-H; **C** Stabilization of a C-7 azole conformation by a favorable azole C-H to azaindole nitrogen H-bonding interaction and a favorable azole nitrogen to azaindole N-H interaction; **D** Unfavorable allylic 1,3-type strain interaction between an azole substituent and the azaindole nitrogen atom offset by a favorable azole nitrogen to azaindole N-H interaction

These observations intensified interest in the 6-azaindole series where optimization evolved to focus on the effect of introducing azole and azine heterocycles at the C-7 position in the context of both the 4-F and 4-OCH_3_-substituted series. In this phase of the discovery initiative, both *C*- and *N*-linked connectivities were explored broadly [56, 62, 65]. The SARs that are compiled in Table 8 reinforced the emerging understanding of the preference for compounds in which the C-7 substituent was able to adopt a conformation in which it was coplanar with the azaindole core heterocycle. The 2-substituted thiazole derivatives **172** and **173** are potent antiviral agents while the thiophene **174** is 2- to 4-fold weaker, an observation that can be attributed to reduced planarity. All three compounds can engage the azaindole C-6 nitrogen atom via the low lying C–S σ* orbital on the electron-deficient sulfur atom while the thiazoles **172** and **173** can H-bond to the azaindole N–H, as depicted in Fig. 8A; however, in this conformation the C-3 H-atom of thiophene **174** will be anticipated to sterically interfere with the indole N–H, as depicted in Fig. 8B [56, 66]. The triazoles **175**–**178** and **1** present H-bond accepting heteroatoms to engage the proximal N–H whilst the C–H of these heterocycles can interact with the C-6 nitrogen atom of the azaindole core, interactions that reinforce a planar topography (Fig. 8C). However, the positioning of the CH_3_ substituent in **179** will cause distortion from planarity due to the introduction of allylic 1,3-type strain, explaining the reduced antiviral potency (Fig. 8D) [57–60]. The 4-fold difference in potency between the pyrazoles **180** and **181** presumably reflects a preference for the topological projection of the CH_3_ substituent that will mimic that of triazole **1**. (Fig. 8C). Pyrazine **182**, the only azine from a much broader series that is included in Table 8, is a potent HIV-1 inhibitor that can engage H-bonding interactions between the pyrazine nitrogen and indole N–H and between the pyrazine C–H and azaindole nitrogen. The pyrazine heterocycle is found in the microbicide candidate **183** (DS003, BMS-599793), a molecule selected for development based on its antiviral and PK properties and which builds on the topical microbicidal activity observed with **153** in macaques [67, 68]. Notably, **183** incorporates a 2-phenyl-2-(piperidin-4-ylidene)acetonitrile as a structural replacement for the piperazine benzamide found in **1**.

**Table 8:** SARs associated with C-4, C-7-disubstituted 6-azaindole derivatives


	R	R^1^	EC_50_ (nM)	CC_50_ (μM)
**172**	OCH_3_		0.02	>150
**173**	F^a^		0.05	8.06
**174**	OCH_3_		0.12	36
**175**	OCH_3_		0.78	>300
**176**	F		0.05	>300
**177**	OCH_3_		0.21	>300
**178**	F		0.07	>300
**1**	OCH_3_		0.14	>300
**179**	F		6.0	>300
**180**	OCH_3_		0.11	>300
**181**	OCH_3_		0.41	>300
**182**	F		0.06	>300


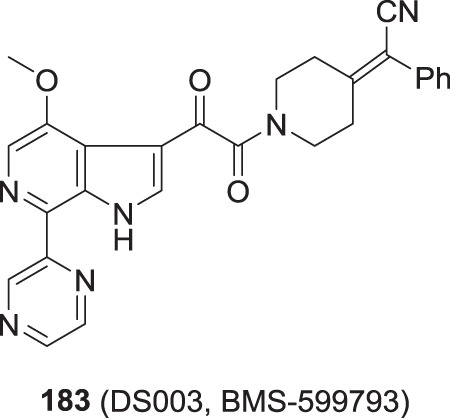

In general, the installation of N-linked azole heterocycles at C-7 offered improved PK properties and additional profiling of **1** and **176** led to their selection as clinical candidates. Both compounds demonstrated an improved antiviral spectrum compared to **154**, with **1** demonstrating potent inhibition of a range of laboratory isolates and, most importantly, recent clinical isolates harvested from the phase 3 clinical trial of the HIV-1 protease inhibitor atazanavir [7, 62, 65, 69–71]. The primary in vitro antiviral potencies and rat PK data for **1** and **176** are compiled in Table 9 [7, 62, 65]. Biochemical pharmacological studies with **1** revealed a more complex binding interaction with HIV-1 gp120 than was observed with **154**, with a biphasic dissociation profile in which the t_1/2_ for the first phase was 7.6 h while that for the second phase was 23 h [7, 69]. The *K*_d_ value measured for **1** was 0.83 ± 0.08 nM which compared to 19 ± 1 nM for **154**, reflecting a 23-fold improvement in affinity [7, 69]. Thus, **1** bound more tightly and with much slower off-rate binding kinetics than **154**.

**Table 9:** Antiviral activity in the pseudotyped assay and rat PK parameters associated with **1** and **176**

	1	176
Pseudovirus EC_50_ (nM)	0.14	0.05
Rat F (%)	82	64
AUC 24 h (μM.h)	111 ± 25	86 ± 33
IV CL (mL/min/kg)	1.3 ± 0.19	1.6 ± 0.2
IV t _1/2_ (h)	4.3 ± 1.1	
V_ss_ IV (L/kg)	0.36 ± 0.098	0.49 ± 0.26

Dosed at 1 mpk IV and 5 mpk PO

At this juncture of the program, clinical studies with **154** were establishing antiviral efficacy in HIV-1 infected patients, with 67% of treated patients experiencing a > 0.5 to 1.5 log_10_ copies/mL decline in viral load with a mean 0.72 log_10_ copies/mL reduction after 800 mg BID and a mean 0.96 log_10_ copies/mL decline after 1600 mg BID [72]. However, in order to sustain the targeted C_min_ at 12 h post-dose, administration of **154** in conjunction with a high fat meal was required. This result was interpreted to be a function of the poor aqueous solubility of **154** underlying inadequate dissolution in the gut, thereby compromising absorption. This clinical feedback was of importance to the discovery team which began to consider phosphate-based prodrugs, drawing upon prior experiences with etoposide phosphate (**184**), the taxane prodrugs phosphates **185** and **186** and fosravuconazole (**187**), the latter sought as an IV-administered agent to support the clinical development program for the parent antifungal agent [73–77]. One interesting observation from the clinical development program for **187** was that it exhibited improved oral bioavailability compared to formulations of the parent drug and it is this prodrug form as the lysine salt that was approved in Japan in January, 2018, where it is marketed as Nailin® for the treatment of onychomycosis, a fungal infection of nails [77, 78]. It was against this backdrop that the potential of phosphonooxymethyl derivatives **188**, **189** and **2** that are derived from **154**, **176** and **1**, respectively, began to be explored as an approach to address the solubility and dissolution issues associated with these HIV-1 AIs [7, 62, 65, 79].
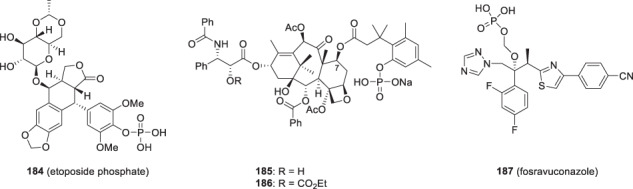


**Scheme 17:**
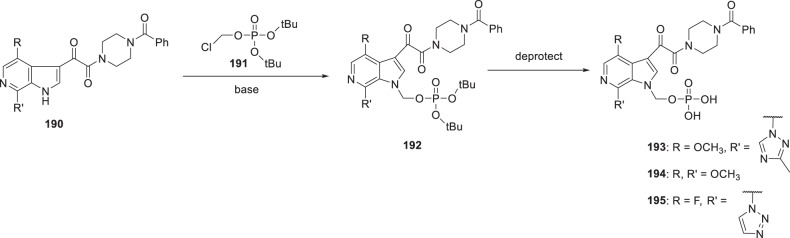
General synthetic approach to phosphonooxymethyl prodrugs **193**–**195**

**Scheme 18:**
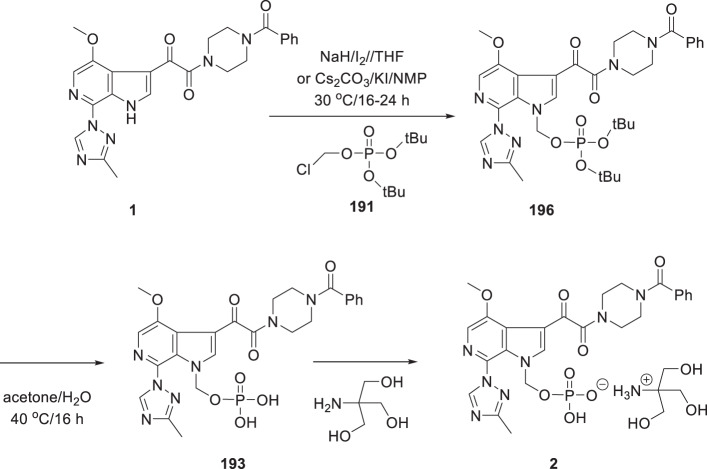
Specific conditions for the synthesis of phosphonooxymethyl prodrug **2**

**Scheme 19:**
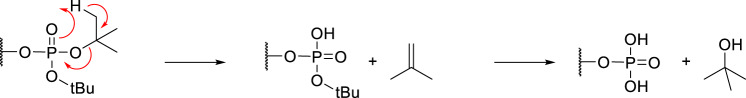
Elimination mechanism for the initial deprotection of the *tert*-butyl phosphate prodrug precursor

Studies with early forms of **2**, **188** and **189** were sufficiently promising that significant effort was expended to develop synthetic approaches and experimental protocols that allowed the isolation of pure and stable crystalline forms of these prodrugs. A careful salt screening exercise identified the lysine salts of **188** and **189** as optimal while for **1** the tromethamine salt was preferred and, after some experimentation to optimize conditions, these prodrugs could be crystallized directly from the reaction mixture in a stable and pure form after completing deprotection of the *tert*-butyl ether synthetic precursors. The phosphonooxymethyl prodrugs **2**, **188** and **189** were prepared by the general approach delineated in Scheme 17 which entailed alkylation of the azaindole N–H of **190** with freshly prepared di-*tert*-butyl chloromethylphosphate (**191**) in the presence of a base to give **192** [62, 65, 79]. While the *tert*-butyl moiety of these intermediates could be removed under acidic conditions, the instability of the free dicacids **193**–**195** presented isolation challenges that were resolved by developing a mild deprotection protocol that was conducted under essentially neutral conditions. The optimal process for deprotection of **192** that was developed was to heat the compound in a mixture of H_2_O and acetone at 40 °C for 16 h which produced a solution of the di-acid to which was added a base at 20 °C, either tris(hydroxymethyl)methylamine (tromethamine) for **193** or lysine for **188** and **189**, and the mixture stirred for 16–24 h before filtering off the crystalline salt of the prodrug. This process required considerable experimentation in order to identify conditions that optimally delivered stable and crystalline salts with the targeted level of purity. The specific conditions employed to secure **2** during the campaign to provide quantities of the prodrug for pre-nomination in vivo toxicology studies are delineated in Scheme 18 [65, 79]. Subsequently, careful studies by the chemical process group revealed that the first *tert*-butyl protecting element is removed within 2 h to generate an acidic product capable of catalyzing cleavage of the second *tert*-butyl moiety [80–82]. In these studies, it was determined that 75% of the *tert*-butyl moiety was released as tBuOH while the remaining 25% was released as *iso*-butylene, the bulk of which was formed in the first 30 min following reaction initiation. These data are consistent with an elimination-type of reaction contributing, in part, to the process of unmasking of the phosphate moiety, as summarized in Scheme 19 [83]. Under these circumstances, a mono-acidic species is released that would promote acid catalyzed degradation of the *tert*-butyl moiety and be autocatalytic in nature. Ultimately, the optimized conditions for large scale prodrug deprotection used a 2:1 mixture of CH_3_CO_2_H and H_2_O at 35 °C to overcome several issues, including solubility problems, material throughput, and product purity [81].
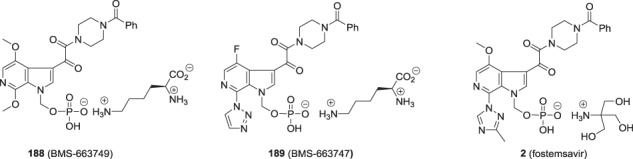

**Scheme 20:**
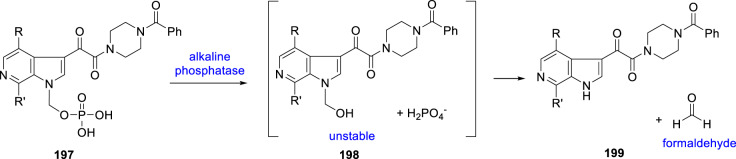
Phosphonooxymethyl prodrug release mechanism

The mechanistic pathway by which the phosphonooxymethyl prodrugs are released in vivo is presented in Scheme 20 and relies upon removal of the phosphate moiety by alkaline phosphatase, a ubiquitous enzyme in vivo that is located in the brush border membrane of the GI tract, to reveal the hydroxymethylated intermediate **198** [84–86]. It is essential for the effective delivery of the parent drug that **198** decomposes spontaneously to release formaldehyde, a process that is favored by the low p*K*_a_ value of the azaindole N–H which was measured as 9.3 for **154**. Under these circumstances, prodrug activation occurs pre-systemically which restricts prodrug exposure to the GI tract while minimizing systemic exposure. A critical element of success with this type of prodrug design is that the parent compound released in this fashion must be a molecule with high membrane permeability so that it is absorbed at least as rapidly as it is produced by the action of alkaline phosphatase at the brush border membrane [84–86]. This is essential in order to prevent parent drug accumulation and precipitation which will frustrate the approach. Since HIV-1 AIs are categorized as biopharmaceutics classification system (BCS) class 2 molecules, this prodrug strategy is well-suited to the in vivo delivery of these compounds [87]. In vitro experiments revealed that the three phosphonooxymethyl prodrugs **2**, **188** and **189** were cleaved rapidly by human alkaline phosphatase to release the parent drug and that they were also cleaved in Caco-2 cells and human hepatocytes; however, they were stable in HLM and plasma from rat, mouse, dog, and cynomolgus monkey. Following IV dosing to rats, dogs and cynomolgus monkeys, all three prodrugs were cleaved rapidly to release their respective parent AIs **1**, **154** and **176**, which were detected as early as 2 min post-dose in rats. Following oral dosing to rats, dogs and cynomolgus monkeys, parent drug appeared rapidly in plasma with only very low levels of prodrug detected and then only at early time points following prodrug administration. While the ability of these phosphonooxymethyl prodrugs to deliver parent AI in vivo at low dose was often comparable to that of dosing a solution of the parent drug, it was in dose-escalation studies that they revealed their clear advantage as a vehicle for drug delivery. Dosing of **188** to the rat and dog confirmed the ability of the prodrug to deliver **154** to plasma in a dose-related fashion while comparison of the clinical formulation of **154** with the prodrug **188** in the dog demonstrated the superiority of the latter, particularly with respect to the absence of a food effect on drug exposure in plasma.

While there were some concerns expressed around the potential for toxicity associated with the release of formaldehyde from these prodrugs, this was placed in perspective, in part, based on the precedent of the marketed prodrug therapeutic agents fosphenytoin (**200**) and tenofovir disoproxil (**201**) which release one and two molecules of formaldehyde, respectively, in vivo. Subsequent to advancing **2** and the lysine salts of **194** and **195** into clinical studies, the issue of formaldehyde release from drugs and prodrugs was addressed more definitively in an insightful published analysis [88]. The quantities of formaldehyde released by 600, 800 and 1200 mg doses of **2** are compiled in Table 10 and amount to 30.66 mgs or 0.44 mpk for a 70 kg individual at the highest dose tested clinically. By way of comparison, the approved clinical doses of **200** and **201** release 103 and 28.1 mgs of formaldehyde, respectively, while the caffeine (**202**) in an 8 ounce cup of coffee can release between 14 and 31 mgs of formaldehyde as a function of demethylative metabolism of the naturally occurring xanthine derivative [88, 89].

**Table 10:** Amount of formaldehyde released in vivo from clinically used doses of **2** and **200**–**202**

	Dose	Amount of formaldehyde released	
**2**	600 mg	15.33	0.22 mpk
	800 mg	20.44	0.29 mpk
	1200 mg	30.66	0.44 mpk
**200**	~1400 mg	103 mg	1.5 mpk
**201**	300 mg	28.1 mg	0.4 mpk
**202**	95–200 mg	14.7–30.9 mg	0.21–0.44 mpk

All mpk data based on 70 kg individual. Data for **202** is based on an 8 oz. cup of coffee that assumes that 80% of the dose is metabolized


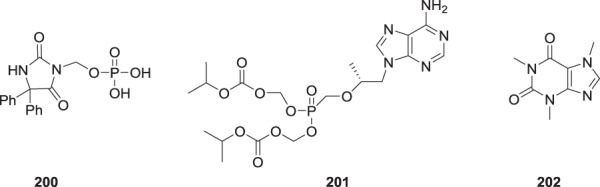


**Scheme 21:**
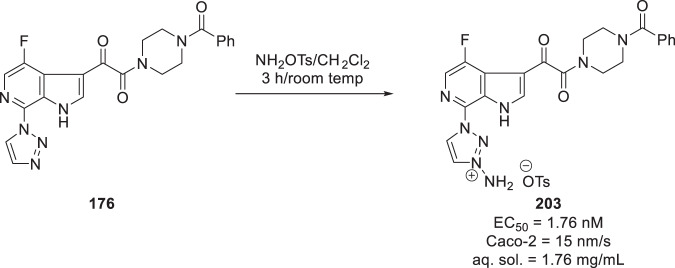
Synthesis of the amminium prodrug of the HIV‑1 attachment inhibitor **176** [90]

An interesting avenue not on the main path for the lead compounds was the discovery of an alternate prodrug approach for the HIV-1 AI **176** in the guise of the charged amminium derivative **203** [90]. Exposure of **176** to *O*-tosylhydroxylamine at room temperature in CH_2_Cl_2_ as the solvent afforded the *N*-amminium derivative **203** in which the site of amination was determined to be the 1,2,3-triazole ring Scheme 21. This molecule offered 250-fold improved aqueous solubility (1.76 mg/mL) compared to the progenitor **176** (0.007 mg/mL) but was 30-fold less potent as an inhibitor of HIV-1 infection in the pseudotyped assay while membrane permeability across a confluent Caco-2 cell layer was 10-fold lower (15 nm/s) than the parent drug. Although *N*-amminium derivatives were not known as prodrugs, by drawing analogy to *N*-oxide prodrugs that can be reduced in vivo by cleavage of the *N*–*O* bond, a series of in vitro and in vivo experiments were conducted in order to assess the potential of **203** to act as a prodrug of **176**. Following IV administration of **203** to rats, **176** was detected in plasma within 5 min but the AUC for the prodrug was 2-fold higher than the parent drug. However, after oral dosing the circumstance was reversed, with the AUC for **176** more than 1350-fold higher than for **203**, indicative of facile prodrug conversion in vivo. The profile of **203** in the dog was similar to the rat, with modest conversion to **176** following IV dosing (the AUC of the parent drug amounted to ~16% of the prodrug) while the ratio was inverted following oral administration with the C_max_ of parent 8-fold higher than that of the prodrug. Most importantly, in a dose escalation study conducted in rats, administration of 5, 25 and 200 mpk of **203** was associated with a linear increase in the plasma exposure of **176**, with the C_max_ values increasing from 1.3 μM to 12 μM and 77 μM, respectively, while the AUC values increased commensurately. In contrast, doses of 15, 75 and 200 mpk of **176** were associated with C_max_ values of 6.6, 8.4 and 11 μM, respectively, a relatively flat dose-exposure relationship that can be attributed to solubility and dissolution issues. While this profile held promise, specifically how and where **203** was cleaved in vivo to **176** remained a conundrum, with no conversion observed in rat blood or plasma or upon incubation in rat liver cytosol, microsomes or hepatocytes under conventional aerobic conditions. However, incubating **203** in RLMs and HLMs under anaerobic conditions resulted in the observation of low levels of conversion to **176**. Further studies revealed that conversion of **203** to **176** was occurring in the GI tract and mediated by the gut microflora, with antibiotic pretreatment reducing the C_max_ and AUC of the parent drug from the prodrug by 5.6- and 3.5-fold, respectively, as summarized in Table 11 [89]. Further development of **203** was not pursued out of concerns for variation in the gut microflora in humans and the potential for drug-drug interactions (DDIs) with antibiotic agents. Nevertheless, the performance of **203** in delivering **176** in dose escalation studies conducted in preclinical species was impressive and two other *N*-amminium-based heterocycles were shown to readily convert to the uncharged parent heterocycles in fresh rat fecal homogenate, suggesting some generality for applications of this kind of prodrug technology [90].

**Table 11:** PK parameters for exposure of **176** after dosing of **203** in the presence and absence of antibiotics

	203		176	
	+ antibiotics	− antibiotics	+ antibiotics	− antibiotics
C_max_ (nM)	79	70	899	5093
t_max_ (h)	0.25	0.25	18	7
AUC_last_ (nM*h)	148	112	13,761	47,535

While the phosphonooxymethyl prodrug **2** delivered **1** to plasma in humans in a dose-related fashion, the efficiency of delivery revealed that the plasma half-life of **1** was prolonged by the phenomenon of flip-flop kinetics, attributed to a prolonged absorption phase due to poor intrinsic solubility of the drug. The solution to this problem was the development of three extended release formulations from which was selected that which favored the release of **1** from **2** in the ascending colon to produce a C_max_/C_min_ ratio of 20 following BID administration at a dose of 600 mg [91, 92]. To assess the efficacy of the extended release form, a phase 2a clinical study in which doses of **2** of 600 mg BID, 1200 mg BID, and 1200 mg QD with ritonavir and 1200 mg BID in the absence of ritonavir were administered to HIV-1 infected subjects for a period of 8 days was conducted [93]. In this trial, all dosed subjects experienced a reduction in plasma viral RNA of >1 log_10_ copies/mL with the maximum median decrease ranging from 1.21 to 1.73 log_10_ copies/mL. Those subjects infected with HIV-1 that demonstrated in vitro susceptibility to **1** with IC_50_ values of less than 100 nM achieved a decline in vial load in excess of 1.5 log_10_ copies/mL. In a phase 2b clinical study, the efficacy of **2** was compared with ritonavir boosted atazanavir where the drugs were used in combination with a 400 mg BIDD dose of the HIV-1 integrase inhibitor raltegravir (RAL) and a 300 mg QD dose of the nucleoside analog TDF (**201**) [94]. The doses of **2** that were evaluated in this study were 400 mg BID, 800 mg BID, 600 mg QD and 1200 mg QD with an eight day monotherapy element in each arm built into the protocol. Across the doses of **2**, there was a mean reduction of 0.7–1.47 log_10_ copies/mL. In these treatment-experienced, HIV-1-infected subjects, the overall response rates were comparable to those treated with the boosted atazanavir drug combination. Through week 24, 78–87% of the subjects administered the drug combination including **2** and 86% of those taking boosted atazanavir had plasma HIV-1 RNA levels of less than 50 copies/mL (observed). The response rates were comparable regardless of the in vitro sensitivity of the virus to **1**. In this study, **2** was generally well tolerated across all arms with no dose-response safety signal and the 600 mg BID dose was selected for the phase 3 clinical studies [94–96]. The phase 3 clinical trial assessed the efficacy of **2** in 371 adults with multidrug-resistant HIV-1 infection over 96 weeks on top of optimized background therapy (OBT) with the subjects divided between randomized (272 subjects who could add **2** or placebo (3:1 ratio) to their failing therapy) and non-randomized (99 subjects who had no remaining antiretroviral options and were administered 2 open-label) cohorts. The primary endpoint was viral load reduction over days 1–8 of a monotherapy period with secondary endpoints defined as the percentage of subjects experiencing a ≥ 0.5 or 1.0 log_10_ copies/mL reduction in viral RNA at day 8 of the monotherapy period, the percentage achieving a viral load of <40 copies/mL at 24, 48, 96 weeks and the mean change in CD4-positive T-cell count through week 96. In the randomized group administered **2**, plasma HIV-1 RNA measured at day 8 of the monotherapy period declined by 0.79 log_10_ copies/mL which compared with a reduction of 0.17 log_10_ copies/mL observed in the placebo group. At week 48, 54% of the subjects in the randomized cohort and 38% in the non-randomized group had a viral load of less than 40 copies/mL with these subjects experiencing a mean increase of 139 and 64 CD4-positive T-cells per cubic millimeter, respectively. The virological failure rate through week 48 of the study in the randomized cohort was 18%, which was comparable to observations in other clinical trials in subjects with multidrug-resistant infection, with 20 of 47 patients who failed exhibiting substitutions in gp120 [96]. In conclusion, in patients with multidrug-resistant HIV-1 infection with limited therapy options, those who received **2** had a significantly greater decrease in plasma HIV-1 RNA levels than those who received placebo during the first 8 days with efficacy sustained through 48 weeks.

## EPILOGUE

**Fig. 9:**
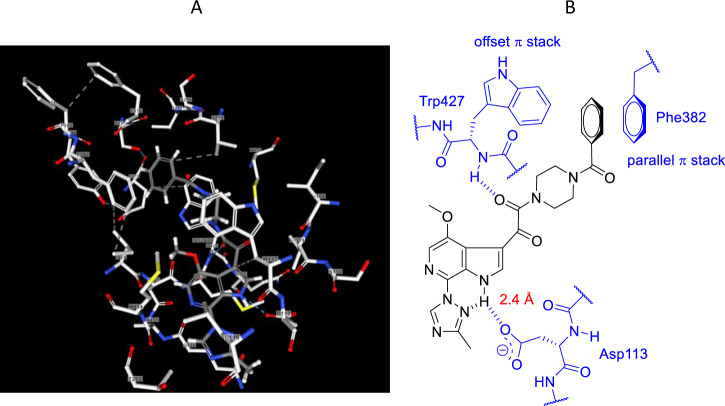
**A** An X-ray cocrystal structure of **1** bound to HIV-1 gp120 (PDB access code 5U7O). **B** Two dimensional plot of the key interactions between **1** and HIV-1 gp120 from an X-ray cocrystal structure

As a first-in-class HIV-1 AI, the optimization of the screening lead **3** into **1** presented significant challenges in drug design while stimulating the development of new synthetic methodologies that allowed an extensive mapping of the pharmacophore by ligand-based design. Insight into drug-target interactions was not obtained until several years after the discovery project had ended, with X-ray cocrystal structure data providing an explanation for the SAR observations that had been painstakingly developed [97, 98]. The AIs were found to bind between the inner and outer domains of gp120 pushing β20-β21 and the Trp427 loop toward the CD4 binding pocket that recognizes Phe43 of the host receptor such that CD4 is prevented from binding, stabilizing the so-called state-1 conformation of the protein in a mechanism that is allosteric in nature [99]. While the drug-target interactions were primarily hydrophobic in nature, the indole N–H established H-bonding interactions with the carboxylate of Asp113 of the protein and a nitrogen atom of the proximal triazole while the benzamide moiety engaged Phe382 and Trp427 in parallel and offset π interactions, respectively, as summarized in Fig. 9 [97, 98].

The exploration of **3** and the challengers that were encountered provided a platform for innovation in synthetic organic chemistry methodology that was fully embraced by creative and inquisitive scientists whose inventions reinforce the symbiotic relationship between the practices of organic chemistry and drug design [8]. However, close collaboration across multiple disciplines in drug discovery and development was an essential ingredient in the successful development of **2** into a marketed product, with considerable additional innovation required to develop a manufacturing process for the active pharmaceutical ingredient (API), a campaign summarized fully in the August 2017 edition of *Organic Process Research and Development* which was devoted entirely to this work [100]. The development of **2** was completed by ViiV Healthcare who began marketing the drug as Rukobia for the treatment of multidrug-resistant HIV-1 infection in the United States in 2020 and in Europe in 2021.

## Abbreviations

ACD: Advanced Chemistry Development
AI: attachment inhibitor
API: active pharmaceutical ingredient
AUC: area under the curve
9-BBN: 9-borabicyclo[3.3.1]nonane
BVDV: bovine viral diarrhea virus
CYP 450: cytochrome P450
DDI: drug-drug interaction
DEPBT: 3-(diethoxyphosphoryloxy)-1,2,3-benzotriazin-4(3*H*)-one
EMA: European Medicines Agency
ELISA: enzyme-linked immunosorbent assay
EmimCl: 1-ethyl-3-methylimidazolium chloride
FDA: United States Food and Drug Administration
GI: gastrointestinal
HIV-1: human immunodeficiency virus-1
HCMV: human cytomegalovirus
HCV: hepatitis C virus
hERG: human ether à go-go-related gene
HLMs: human liver microsomes
HTE: heavily treatment-experienced
IND: investigational new drug
IV: intravenous
MuLV: murine leukemia virus
NaHDMS: sodium bis(trimethylsilyl)amide
nBuLi: n-butyllithium
NHVs: normal healthy volunteers
OBT: optimized background therapy
PEG: poly(ethylene glycol)
PK: pharmacokinetic
RSV: respiratory syncytial virus
SAD: single ascending dose
SARs: structure-activity relationships
RAL: raltegravir
RLMs: rat liver microsomes
SAR: structure-activity relationship
SIV: simian immunodeficiency virus
TDF: tenofovir disoproxil
THF: tetrahydrofuran
VSV: vesicular stomatitis virus
